# Unclassified Diffuse Ductal Cholangiocarcinoma; Report of a Case

**DOI:** 10.1155/2014/542849

**Published:** 2014-03-04

**Authors:** Ünal Aydın, İsmail Özsan, Türker Karabuğa, Özcan Alpdoğan, Ragıp Ortaç, Ömer Yoldaş, Erkan Şahin

**Affiliations:** ^1^Department of Surgery, İzmir University Faculty of Medicine, Turkey; ^2^Department of Pathology, İzmir University Faculty of Medicine, Turkey; ^3^Department of Radiology, İzmir University Faculty of Medicine, Turkey

## Abstract

Cholangiocarcinoma (CCA) is the second most common malignant tumor of the liver. It is simply classified as intrahepatic and extrahepatic CCA (including perihilar and distal extra hepatic CCA) according to the anatomic localization. Various classification systems were described for staging cholangiocarcinoma. We represent an interesting case of cholangiocarcinoma which is in the shadow area of classification by involving intrahepatic, hilar, and distal extra hepatic bile ducts. To our knowledge, this is the first case in the literature with diffuse bile duct involvement.

## 1. Introduction

Cholangiocarcinoma is the second most common primary tumor of the liver. Cholangiocarcinomas are classified according to their anatomic location as intrahepatic and extrahepatic. The anatomic margins for distinguishing intra- and extrahepatic cholangiocarcinomas are the second order bile ducts. Therapeutic modalities vary according to the localization of the tumor. Extrahepatic cholangiocarcinomas can further be subdivided according to the Bismuth classification into types I to IV. As in Bismuth classification various terminologies and classifications have been used to describe the pathologic and radiologic appearance of cholangiocarcinoma and each describes a specific aspect of the tumor. The Liver Cancer Study Group of Japan proposed in 2000 a new classification based on growth (morphologic) characteristics being identified as mass forming, periductal-infiltrating, and intraductal-growing types [1].

Characteristically skipping involvements towards the biliary tract should be observed in cholangiocarcinoma. Here we represent a case which remains unclassified anatomically with the involvement of intrahepatic, perihilar, and distal extrahepatic bile ducts and the patients' intraoperative management.

## 2. Case

### 2.1. Preoperative Workup

**Figure 1:**
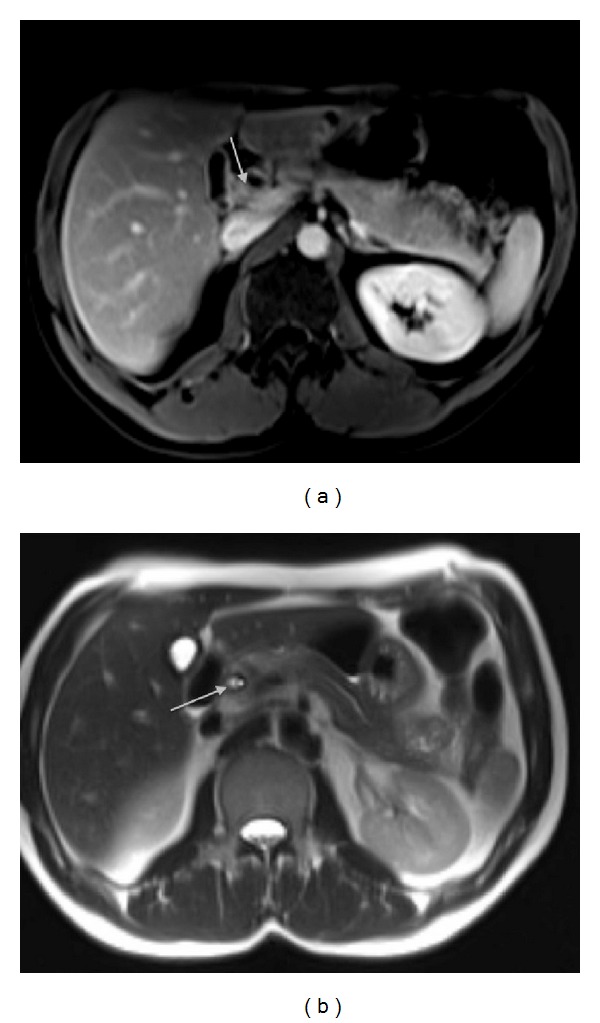
(a) T1 weighted axial postcontrast MRI, asymmetric contrast-enhancing lesion in distal choledoch, and indentation to portal vein (white arrow) ans (b) T2 weighted axial MRI, mild wall thickness in distal choledoch (white arrow).

A 36-year-old man was referred to our hospital with a diagnosis of obstructive jaundice due to distal cholangiocarcinoma. On admission he had a history of jaundice, itching, and 10 kg loss in weight. Regarding biochemical analyses, aspartate aminotransferase level was 100 U/L, alanine aminotransferase was 106 U/L, alkaline phosphatase was 176 U/L, gamma-glutamyl transpeptidase was 134 U/L, and bilirubin level was 1,7 mg/dL. Abdominal ultrasonography revealed minimal choledochal dilatation. Asymmetric contrast-enhancing lesion in distal choledoch, indentation to portal vein, and minimal wall thickness were observed in abdominal magnetic resonance imaging (Figures 1(a) and 1(b)). Endoscopic retrograde cholangiopancreatography (ERCP) revealed a 2 cm segmental stenosis in the middle part of the choledoch and a plastic stent was placed. Cytologic examination of the brush biopsy was suspicious of malign process. The patient was prepared to surgery with the suspectful diagnosis of distal cholangiocarcinoma.

### 2.2. Intraoperative Process

**Figure 2:**
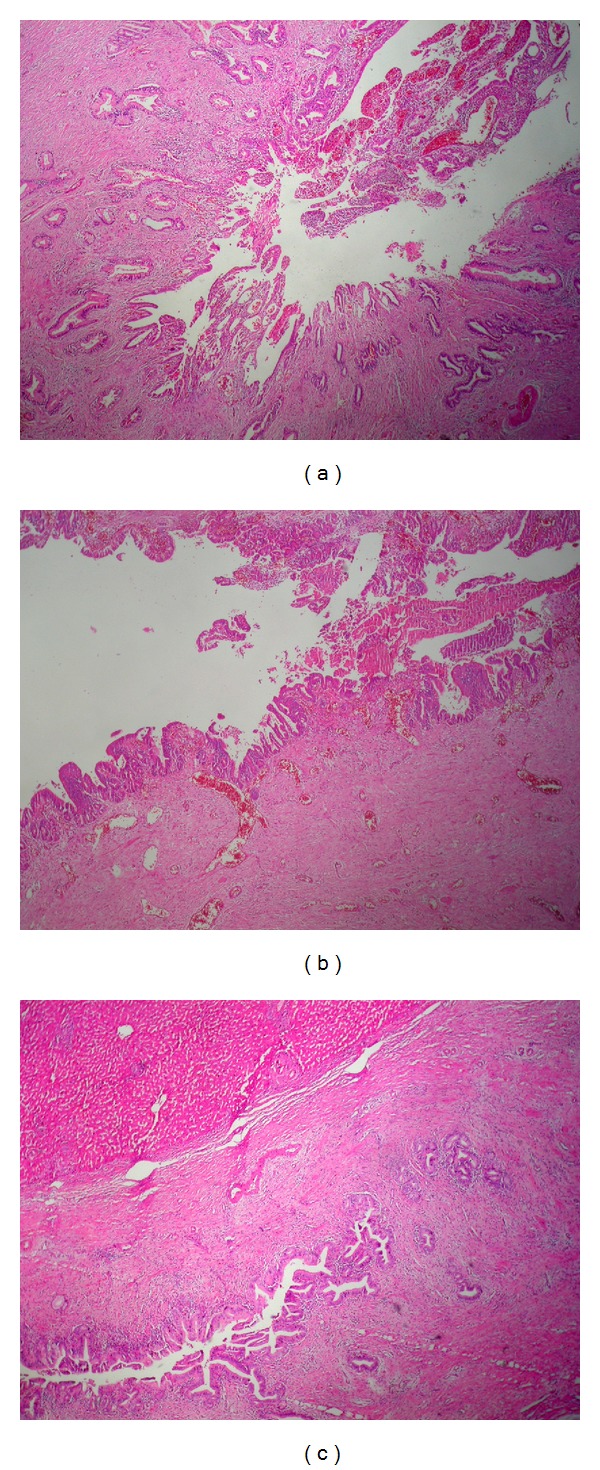
(a) Invasive adenocarcinoma in choledoch (×40 HE), (b) in situ cholangiocarcinoma in left bile duct (×100 HE), and (c) in situ cholangiocarcinoma in left hepatic lobe (×40 HE).

Preoperative surgical plan was radical pancreaticoduodenectomy (Whipple procedure), and the procedure was carried out but surgical margin was not cancer-free in frozen pathological examination (Figure 2(a)), bile ducts were dissected longitudinally towards the bifurcation, and high hilar dissection was made up according to our own technique which was described and shared with the medical literature [2].

Intraoperative ultrasound was achieved and wall thickness and irregularity on left hepatic branches of the bile duct were observed and frozen pathological examination of the resected specimen revealed tumor cell on the left bile duct (Figure 2(b)) included by hepatic segments 2, 3, and 4 (Figure 2(c)). Left hepatectomy and biliary reconstruction was carried out.

**Figure 3:**
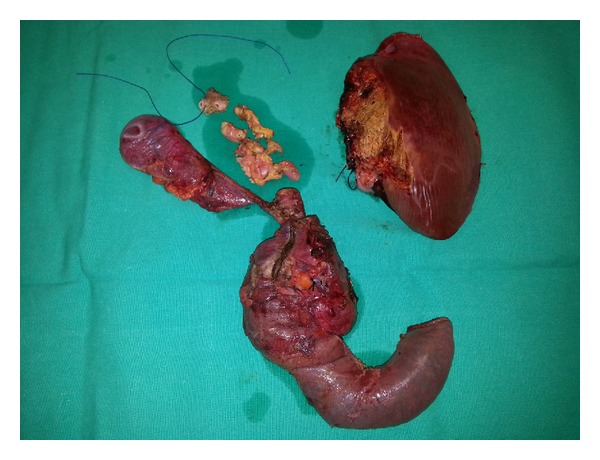
Pancreaticoduodenectomy material, high hilar resection specimens, and left lobe of the liver.

The resected specimens including pancreaticoduodenectomy material, liver, and high hilar bile ducts are shown in Figure 3. Postoperative period was uneventful; the patient was discharged on postoperative sixth day.

## 3. Discussion

Cholangiocarcinoma is a malignant tumor that arises from biliary epithelium at any part of the bile duct system, from bile ductules to ampulla of Vater [3, 4]. The prognosis of this malignancy is dismal owing to its silent clinical character, difficulties in early diagnosis, and limited therapeutic approaches. Tumoral staging should be performed before and after surgery including all intraoperative information from macroscopic and microscopic examination.

In the radiologic literature, hilar and extrahepatic cholangiocarcinomas have been classified as exophytic, infiltrating, and polypoid (or papillary) [5]. The Bismuth-Corlette classification provides preoperative assessment of local spread [6, 7]. The Liver Cancer Study Group of Japan proposed a new classification based on growth characteristics [1]. Several staging systems are described but the majority of these have proven to be insufficient for stratifying patients in alignment with therapeutic options.

These tumors should be treated by different surgical modalities according to the localization of the tumor. Studies suggest that the invasion longitude of hilar cholangiocarcinoma along the bile duct varies significantly with distances ranging from a few millimeters to several centimeters [8].

Skipping involvements could be observed along the bile duct. A positive bile duct resection margin is correlated with higher local recurrence rate and poor prognosis and its role is similar to a positive lymph node [9]. Intraoperative frozen pathological examination of the resected bile duct margin is an important method to determine the modality of the surgical procedure.

To our knowledge, this was the first case in the literature with the diffuse involvement of the bile duct from choledoch to the tertiary ductules of left hepatic lobe.

## References

[1] Liver Cancer Study Group of Japan (2000). *The General Rules for the Clinical and Pathological Study of Primary Liver Cancer*.

[2] Aydin U, Yedibela S, Yazici P (2008). A new technique of biliary reconstruction after “high hilar resection” of hilar cholangiocarcinoma with tumor extension to secondary and tertiary biliary radicals. *Annals of Surgical Oncology*.

[3] Callea F, Sergi C, Fabbretii G, Brisigoiti M, Cozzutto C, Medicina D (1993). Precancerous lesions of the biliary tree. *Journal of Surgical Oncology*.

[4] Nakanuma Y, Minato H, Kida T, Terada T, Tobe T, Kameda H, Okudaira M, Ohto M (1994). Pathology of cholangiocellular carcinoma. *Primary Liver Cancer in Japan*.

[5] Lim JH (2003). Cholangiocarcinoma: morphologic classification according to growth pattern and imaging findings. *American Journal of Roentgenology*.

[6] Bismuth H, Corlette MB (1975). Intrahepatic cholangioenteric anastomosis in carcinoma of the hilus of the liver. *Surgery Gynecology and Obstetrics*.

[7] Bismuth H, Nakache R, Diamond T (1992). Management strategies in resection for hilar cholangiocarcinoma. *Annals of Surgery*.

[8] Lim JH (2003). Cholangiocarcinoma: morphologic classification according to growth pattern and imaging findings. *American Journal of Roentgenology*.

[9] Sasaki R, Takeda Y, Funato O (2007). Significance of ductal margin status in patients undergoing surgical resection for extrahepatic cholangiocarcinoma. *World Journal of Surgery*.

